# Treatment of Infections Due to *Aspergillus terreus* Species Complex

**DOI:** 10.3390/jof4030083

**Published:** 2018-07-09

**Authors:** Cornelia Lass-Flörl

**Affiliations:** Division of Hygiene and Medical Microbiology, Medical University of Innsbruck, Schöpfstraße 41, 6020 Innsbruck, Austria; Cornelia.lass-floerl@i-med.ac.at

**Keywords:** *Aspergillus terreus*, amphotericin B, resistance, antifungal treatment

## Abstract

The *Aspergillus terreus* species complex is found in a wide variety of habitats, and the spectrum of diseases caused covers allergic bronchopulmonary aspergillosis, *Aspergillus* bronchitis and/or tracheobronchitis, and invasive and disseminated aspergillosis. Invasive infections are a significant cause of morbidity and mortality mainly in patients with hematological malignancy. The section *Terrei* covers a total of 16 accepted species of which most are amphotericin B resistant. Triazoles are the preferred agents for treatment and prevention of invasive aspergillosis. Poor prognosis in patients with invasive *A. terreus* infections seems to be independent of anti-*Aspergillus* azole-based treatment.

## 1. Introduction

Aspergillosis refers to infection by any of the Aspergillus species; the most common pathogens are representatives of the *Aspergillus fumigatus* species complex, *Aspergillus flavus* species complex, *Aspergillus terreus* species complex, and *Aspergillus niger* species complex. The *A. terreus* species complex is found in a wide variety of habitats, soil, compost, or dust, and the spectrum of diseases caused covers allergic bronchopulmonary aspergillosis, Aspergillus bronchitis and/or tracheobronchitis, and invasive and disseminated aspergillosis [1,2]. The section Terrei covers *A. terreus sensu stricto* (*s.s.*), *A. alabamensis*, *A. allahabadii*, *A. ambiguus*, *A. aureoterreus*, *A. bicephalus*, *A. carneus*, *A. citrinoterreus*, *A. floccosus*, *A. iranicus*, *A. hortai*, *A. microcysticus*, *A. neoafricanus*, *A. neoindicus*, *A. niveus*, and *A. pseudoterreus*, giving a total of 16 accepted species [2]. Infections due to the *A. terreus* species complex occur worldwide. However, there are certain geographic locations with frequent occurrences, such as Innsbruck, Austria and Houston, USA [3]. Within the aspergilli, the *A. terreus* species complex assumes a specific role, as most representatives are amphotericin B resistant [4]. *A. terreus* is an emerging opportunistic fungus whose clinical incidence has increased in recent years, with invasive diseases constituting nearly 4% of all invasive aspergilloses [5]. Of special concern is the high mortality in disseminated diseases caused by this species [6]. Here, we review in vitro susceptibility, experimental and clinical outcome data, and overview recommendations on how to treat infections due to the *A. terreus* species complex.

## 2. The Pathogen and Fungal Epidemiology

**Figure 1:**
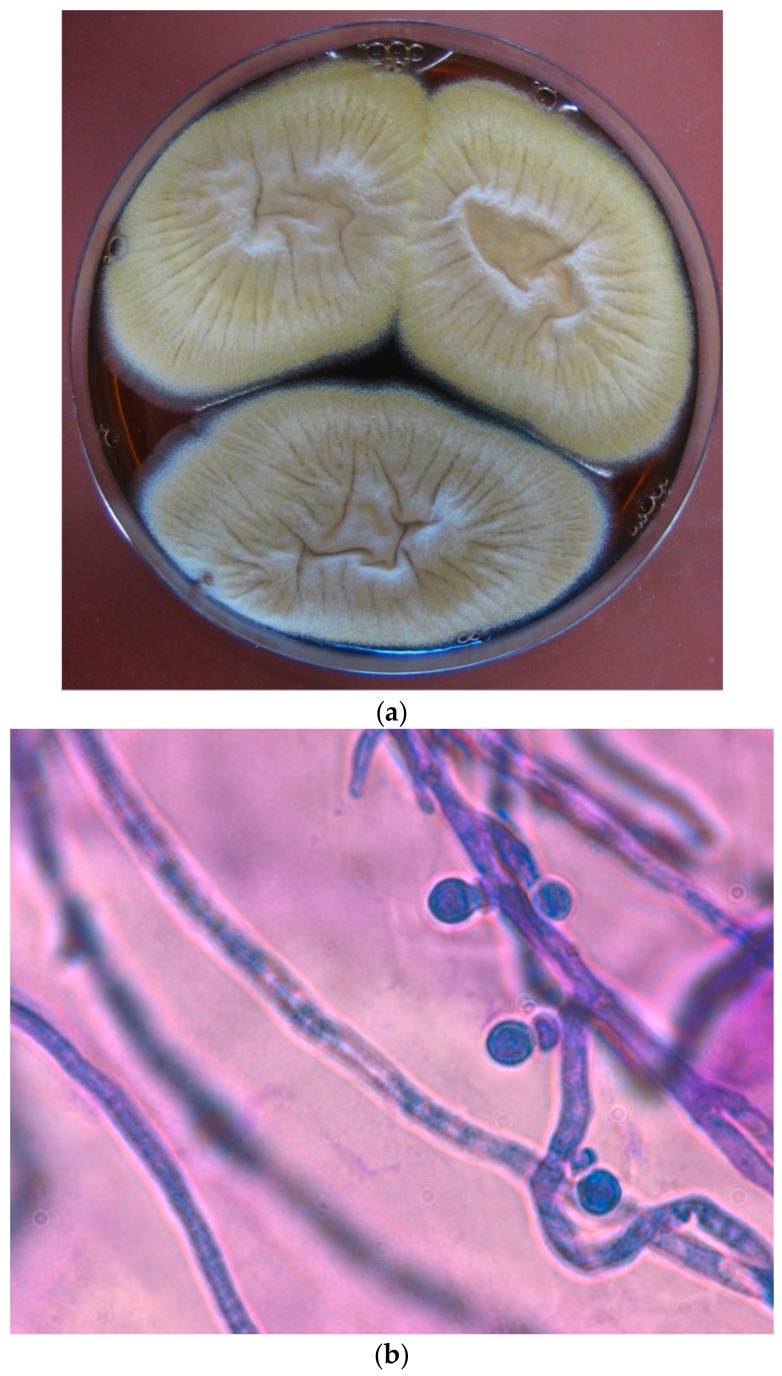

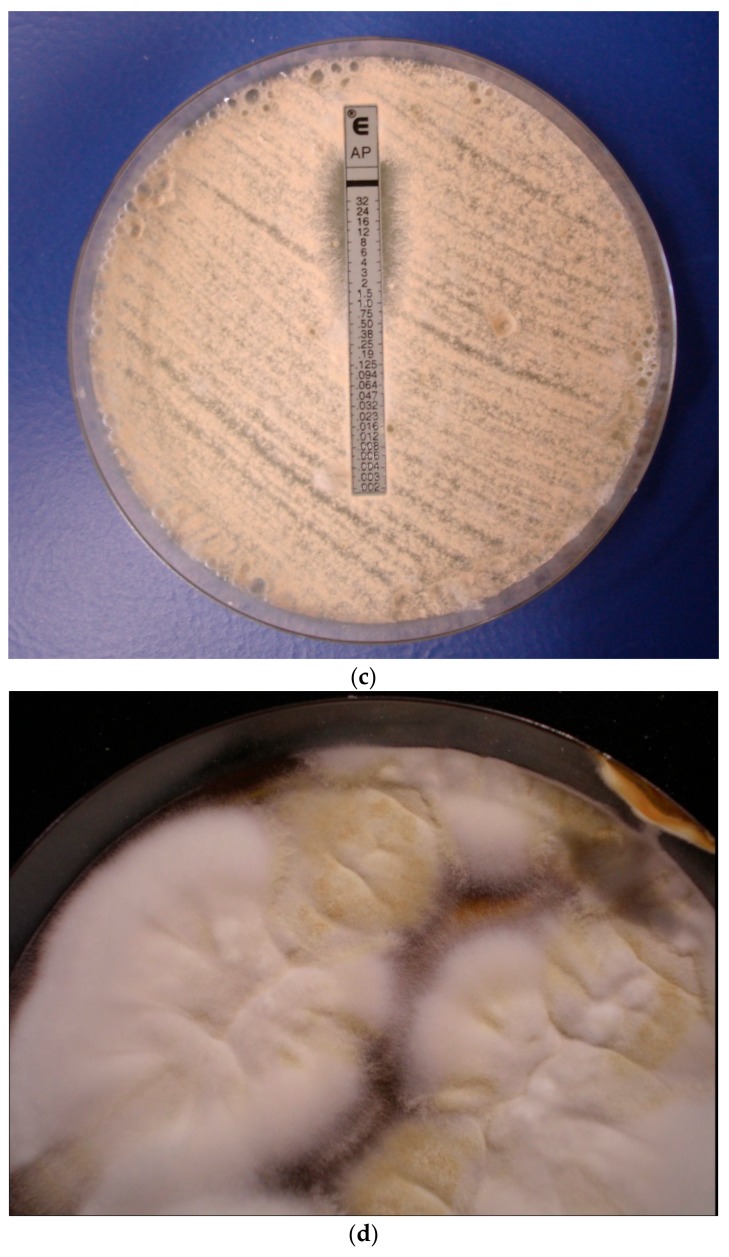
(**a**–**d**) *A. terreus sensu strictu* displaying typical culture morphology on Sabouraud Agar (48 h/37 °C) (**a**) and wet mount preparation with lactophenol cotton blue showing aleuroconidia (light microscopy, magnification ×1000) (**b**), E-Test^®^ showing amphotericin B resistance (**c**) and the performance of sectoring by the production of white, fluffy, and conidia-sparse colonies (**d**).

*Aspergillus* section *Terrei* is widespread throughout the world and found in arable soil, desserts, and compost heaps [2]. *A. terreus* usually shows cinnamon-brown colonies when growing on Saubouraud Agar at 37 °C for 48 h (see Figure 1a). The production of aleurioconidia in *A. terreus*, *A. carneus*, *A. flavipes*, and *A. niveus* seems to be unique among the *Aspergillus* species; these are morphologically distinct lateral conidia (aleurioconidia) which are attached directly to hyphae (see Figure 1b). Whether these features support dissemination or enhanced virulence in vivo is presently unknown. Deak et al. [7] showed that these accessory conidia induce inflammation in a pulmonary mouse model of invasive aspergillosis; amphotericin B resistance was shown not to be associated with the production of aleurioconidia. *A. terreus* is commonly used in industry to produce organic acids and lovastatin. In a prospective international multicenter surveillance study, including 21 countries and 38 centers, an overall prevalence of 5.2% (370/7116) of *A. terreus* infections among mold infections was detected [8]. Cases were reported from Europe (*n* = 261), Middle East (*n* = 70), India (*n* = 19), South America (*n* = 10), and North America (*n* = 10). Spain and Austria were the countries with the highest density of *A. terreus* isolates collected in patients [8].

The reason for this specific epidemiological situation is unclear. *A. terreus* strains isolated from environmental and clinical sources were genotyped using a novel panel of short tandem repeats [9]. Three major endemic genotypes collected from the Inn region and its side valleys were found to cause the majority of invasive *A. terreus* diseases in Tyrol, Austria. The genotypes were of the same mating type and persisted for at least 11 years.

## 3. Patients at Risk Suffering from *Aspergillus terreus* Species Complex

The at risk population for invasive infections due to *Aspergillus* section *Terrei* does not differ from patients suffering from *A. fumigatus* diseases, and comprises mainly individuals with prolonged neutropenia, allogeneic hematopoietic stem cell transplant (HSCT), solid organ transplant (SOT), inherited or acquired immunodeficiencies, and corticosteroid use [6,10]. The intensity and duration of neutropenia predict the main risk of infection, including patients with refractory or relapsed acute leukemia treated with reinduction regimens. However, the *A. terreus* species complex also affects non-immunocompromised individuals with prior or current lung disease [8]. Chronic pulmonary aspergillosis is an uncommon and problematic disease thought to affect ~240,000 people in Europe. A world-wide, prospective study showed *A. terreus* to be involved in various entities, such as chronic lung diseases (*n* = 149, 39.2%), hematologic or oncologic malignancies (*n* = 30, 7.4%), solid organ transplantation (*n* = 30, 7.4%), diabetes mellitus (*n* = 24, 6.6%), solid tumors (*n* = 19, 5.1%), heart (*n* = 24, 6.2%), renal (*n* = 14, 3.7%), and liver diseases (*n* = 4, 1.1%), and others. Underlying diseases comprised invasive aspergillosis (*n* = 93, 25.1%), allergic broncho-pulmonary aspergillosis (*n* = 46, 12.4%), chronic aspergillosis (*n* = 42, 11.4%), chronic obstructive pulmonary disease exacerbation (*n* = 20, 5.5%), aspergilloma (*n* = 14, 3.7%), otitis externa (*n* = 9, 2.5%), wound infections (*n* = 3, 0.7%), and others [8].

## 4. Treatment Recommendations

The effective management of invasive aspergillosis includes strategies to optimize prevention and early antifungal treatment, immunomodulation, and in some cases, surgery. Overall, treatment strategies should be based on the institutional epidemiology of infections and assessment of individual risk factors.

Triazoles are the preferred agents for treatment and prevention of invasive aspergillosis in most patients [11]. Patients receiving triazole-based therapy for treatment of invasive diseases or prolonged prophylaxis should undergo therapeutic drug monitoring (TDM). This approach supports enhancing therapeutic efficacy of voriconazole and posaconazole. Echinocandins have been shown to be effective only in salvage therapy. Amphotericin B is not recommended; however, in selected cases [12,13], improved treatment outcome was observed under high dose amphotericin B treatment. Combinations of polyenes or azoles with echinocandins suggest additive or synergistic effects in some preclinical studies [14,15]. However, variable assay conditions and conflicting results have led to uncertainty as to how to interpret these findings. Early initiation of adequate antifungal therapy is warranted in addition to a diagnostic evaluation [11]. Treatment should be continued for a minimum of 6–12 weeks [11], depending on degree and duration of immunosuppression and the site of infection. Patients with chronic aspergillosis and either pulmonary or general symptoms or progressive loss of lung function should be treated for a minimum of 6 months. Oral itraconazole and voriconazole are the preferred drugs [11]; posaconazole is another option for individuals with adverse events or clinical failure. Hachem et al. investigated 513 patients suffering from hematological malignancies and compared baseline characteristics, antifungal treatment, and outcome between patients infected with *A. terreus* and non-*terreus Aspergillus* species [10]. *A. terreus* diseases were associated with a lower response rate to treatment, and a higher rate of mortality when compared to non-*terreus* infections. In addition, fungal breakthrough infections occurred more often in the *A. terreus* arm; independent factors associated with final outcome included treatment with azoles (*p* < 0.0001) and *Aspergillus* species involved (*p* = 0.043).

## 5. Amphotericin B

Amphotericin B, a polyene antifungal drug, was developed in the 1950s and is used intravenously to treat invasive aspergillosis, cryptococcal meningitis, candidiasis, and mucormycosis. In vitro resistance to amphotericin B is rare; few fungal species such as *A. terreus*, *Aspergillus tanneri, Fusarium* species, and *Scedosporium prolificans* show polyene resistance [16,17]. The major clinical interest in *A. terreus* infections is related to its amphotericin B resistance (see Figure 1c); retrospective studies revealed high rates (80–90%) of treatment failure with amphotericin B [18]. The Clinical Laboratory Standard Institute (CLSI) and the European Committee on Antimicrobial Susceptibility Testing (EUCAST) have categorized *A. terreus* as resistant to amphotericin B [17,19]. Furthermore, 70% of *A. terreus* isolates investigated showed minimum fungicidal concentrations (MFCs) to be twofold higher than the obtained minimum inhibitory concentrations (MICs). With these findings, the authors underline the poor efficacy against *A. terreus* infections, specifically in neutropenic patients [15]. Animal studies confirmed resistance of *A. terreus* infections even to high doses of amphotericin B or its liposomal formulation in murine and rabbit models. However, a single experimental study in neutropenic mice suggested *A. terreus* to be susceptible to high doses of liposomal amphotericin B, achieving high concentrations in the lungs (4–8 mg/L) [13]. *A. terreus* is intrinsically resistant to amphotericin B, and the underlying mechanisms may be associated with the level of catalase production by this species when compared to *A. fumigatus* [20,21,22]. Studying amphotericin B resistant (ATR) and amphotericin B susceptible (ATS) clinical *A. terreus* isolates showed ATR to possess doubled basal superoxide dismutase (SOD) activity when compared to ATS; in addition, ATR presented an enhanced oxidative stress response (OSR), with significantly higher *sod2* mRNA levels and increased *catalase* transcripts during amphotericin B treatment. The inhibition of SOD and catalase (CAT) proteins rendered ATR to ATS in vitro [20,21,22].

Hypoxia seems to have a major influence on the activity of amphotericin B against *A. terreus* [23]. A significant decrease in the MICs against amphotericin B was detected, mainly due to the loss of mycelium sterillium zones applying the Etest^®^. In addition, the fungicidal activity of amphotericin B against *A. terreus* was significantly decreased under hypoxia, suggesting a shift to fungistatic activity under low-oxygen conditions. Whether these findings have any clinical relevance needs to be investigated in more detail. The alternative in vivo model *Galleria mellonella* was applied to study the pathogenicity of ATS and ATR, and to evaluate amphotericin B efficacy in vivo [24]. Larvae were susceptible to *A. terreus* infection in an inoculum-size and temperature dependent manner, and in vitro susceptibility to amphotericin B correlated with in vivo outcome, defining an amphotericin B susceptible strain cluster of *A. terreus* [24]. Usually, the MICs of amphotericin B are generally ≥2 mg/L (see Figure 1c), but low MICs (0.06–0.125 mg/L) have also been reported for the *A. terreus* species complex. To our knowledge, the real origin of ATS is unknown; ATS may exist in the environment or may reflect an accidental product of sectoring of ATR on drug-free medium, or may result from both [25]. In a clinical setting, ATS occurs rarely, and we have shown that spontaneous culture degeneration of ATR leads to the emergence of multiple sectors (see Figure 1d), which in turn displayed low MICs against amphotericin B, hence featuring ATS. Susceptibility and virulence studies of sector subcultures (ATSec), ATR, and ATS strains showed ATSec harbouring amphotericin B MICs ranging from 0.12 to 0.5 μg/mL, and *G. mellonella* survival studies revealed an enhanced virulence of ATSec [25]. With regard to antifungal susceptibility testing, this is a very important finding; any colony selected for inoculum preparation should be free of sectoring. Otherwise, MIC data may be unstable in repeating tests, further complicating the interpretation of MIC and in vivo outcome. We noticed that sectoring frequently takes place on various culture media in ATR.

## 6. Azoles

Voriconazole is the drug of choice for first-line treatment of invasive aspergillosis including *A. terreus* [26]. Limited data are available for itraconazole, posaconazole, and isavuconazole. Four (44%) out of nine patients were treated with posaconazole (salvage therapy) and responded favorably, whilst the success rate of lipid amphotericin B formulations was only 19%. There is limited clinical experience on the efficacy of the echinocandins in the treatment of *A. terreus* infections. A murine model of *A. terreus* disseminated infection, with isolate MICs ranging from 0.12 mg/L to 4 mg/L in evaluated voriconazole treatment [27]. Improved survival and a reduced fungal load in brain and kidney were given in strains displaying MICs ≤1 mg/L. Few experimental data showed that high itraconazole doses may be effective in the treatment of animals infected with *A. terreus*. Posaconazole has shown efficacy in treating invasive infections by *A. terreus* in rabbits and mice.

Recently, a considerable amount of azole resistance in *Aspergillus* section *Terri* was observed; however, whether the latter finding correlates with in vivo results is presently unknown [28]. The emergence of *A. terreus sensu lato* (*s.l.*) isolates with reduced azole-susceptibility was reported in patients suffering from cystic fibrosis [29]. In a worldwide study performed, approximately 5% of all tested *A. terreus s.s.* isolates were resistant to posaconazole in vitro [28]. In Austria, Germany, and the UK, posaconazole resistance was higher than 10% in all *A. terreus s.s.* isolates. The majority of isolates was identified as *A. terreus* (86.8%), followed by *A. citrinoterreus* (8.4%), *A. hortai* (2.6%), *A. alabamensis* (1.6%), *A. neoafricanus* (0.2%), and *A. floccosus* (0.2%). Posaconazole resistance differed geographically and ranged from 0% in the Czech Republic, Greece, and Turkey, to 13.7% in Germany. In contrast, azole resistance among cryptic species was rare, and was observed only in one *A. citrinoterreus* and one *A. alabamensis* isolate. Resistance against itraconazole and voriconazole was not detected [28]. Azole resistance in *A. terreus s.s.* and *A. fumigatus* is associated with mutations and alterations of the lanosterol-14-α steroldemethylase gene (*Cyp51A*), a key protein in the ergosterol biosynthesis pathway [30]. However, aside from mutations in the primary target gene, other less known mechanisms (e.g., efflux pumps, overexpression of *cyp51*) were also found to be involved in azole resistance.

## 7. Conclusions

**Figure 2:**
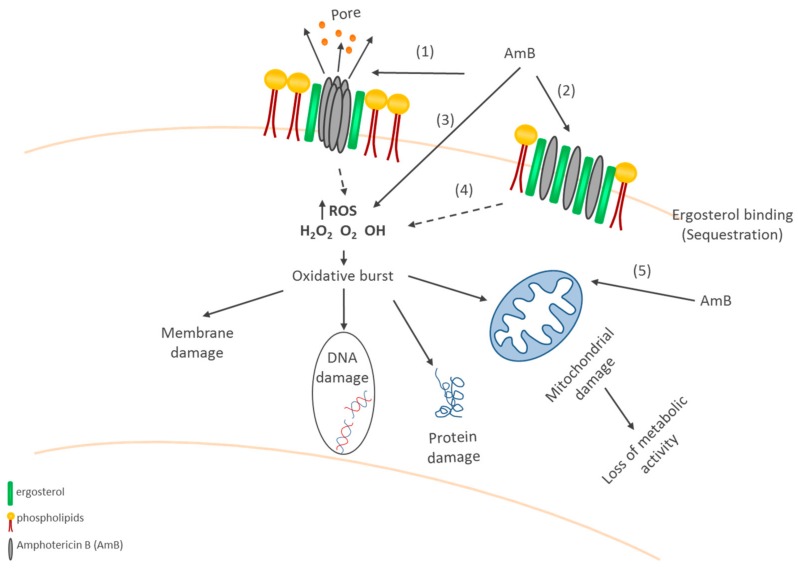
Amphotericin B: Mode of action on *A. terreus*. There exist two major mechanisms of amphotericin B (AmB): pore formation in fungal cell membranes (1,2,3) and induction of oxidative stress (3,4,5) causing cell permeability and cell death. Amphotericin B may act as an oxidant as well as antioxidant [22]. Several AmB molecules penetrate the cell, where oxidation takes place, and radicals emerge.

The effective management of invasive aspergillosis includes strategies to optimize prevention and early antifungal treatment, immunomodulation, and in some cases, surgery. Overall, treatment strategies should be based on the institutional epidemiology of infections and assessment of individual risk factors. Triazoles (Vorriconazole and isavuconazole) are the preferred agents for the treatment and prevention of invasive aspergillosis due to *A. terreus* in most patients [11]. The underlying resistance mechanisms of *A. terreus* against amphotericin B are only partly understood. The mode of action of amphotericin B seems to be complex and multifaceted (see Figure 2); amphotericin B interacts in various ways with ergosterol, and hence results in fungal cell membrane disruption leading to intracellular leakage and cell death [22]. In addition, amphotericin B may directly act as a pro-oxidant or anti-oxidant, and contribute to an accumulation of free radicals which have multiple deleterious effects. We demonstrated that the co-application of anti- and pro-oxidants significantly affects amphotericin B efficacy in an antithetic manner [21]. Antioxidants and ROS-scavenging agents increased amphotericin B tolerance in susceptible *A. terreus* strains, while pro-oxidants rendered them resistant to susceptible strains; this in vitro data was confirmed in vivo by applying *Galleria mellonella* animal models. Whether such management is of clinical interest needs to be studied in detail.
